# Cross-Sectional Study of Seroprevalence and Associated Risk Factors of Bovine Brucellosis in Selected Districts of Jimma Zone, South Western Oromia, Ethiopia

**DOI:** 10.1155/2022/9549942

**Published:** 2022-06-25

**Authors:** Monenus Etefa, Tadele Kabeta, Desalegn Merga, Motuma Debelo

**Affiliations:** ^1^Ilu Livestock Resource Development Office, Teji, Oromia, Ethiopia; ^2^Jimma University, College of Agriculture and Veterinary Medicine, Jimma, Oromia, Ethiopia; ^3^Bedelle Regional Veterinary Laboratory Center, Bedelle, Oromia, Ethiopia

## Abstract

Bovine brucellosis is one of the most widespread but neglected zoonotic diseases in developing countries where it is an endemic and growing problem causing public health impacts. Developing a cost-effective control strategy of the disease can only be guaranteed by knowledge of the disease epidemiology that defines its risk profiles. Hence, this study was designed to evaluate epidemiological aspects of bovine brucellosis in selected districts of Jimma zone. A cross-sectional study with multistage sampling techniques was conducted on 424 cattle to evaluate its seroprevalence. Likewise, 114 households were included for the investigation of risk factors. SPSS version 20 for data analysis and C-ELISA test for antibody detection were used. Moreover, the chi-square test for univariable analysis and logistic regression model for multivariable analysis were employed to assess association between seropositivity and risk factors. From this study, 3.3% (95% CI: 1.82-5.48) and 12.3% (95% CI: 6.88-19.75) seroprevalence of the disease was detected with the highest proportion found at Kersa district (6.5 (95% CI: 1.37-17.90) and (21.4 (95% CI: 4.66-50.80)) followed by Seka Chokorsa (1.76 (95% CI: 0.37-5.07) and (6.7 (95% CI: 1.40-18.27)) and Mana (1.75 (95% CI: 0.21-6.20) and (7.1 (95% CI: 0.88-23.50)) at individual animals and herd levels, respectively. Cattle of poor body condition, pregnant, and cows with history of abortion and repeat breeding were found 4.8 (95% CI: 2.00-22.74), 4.3 (95% CI: 1.43-13.04), 3.3 (95% CI: 1.07-10.21), and 2.7 (95% CI: 1.86-8.15) times more likely seropositive than their counterparts, respectively. Besides these, mixed feeding style was highly associated with seropositive reactors than separate feeding (AOR = 8.3; 95% CI: 1.76-38.99). These findings depicted substantial areas to be addressed in implementation of appropriate and immediate control actions and establishment of intervention mechanisms of bovine brucellosis.

## 1. Background

Brucellosis is one of the oldest and most widespread zoonotic diseases, affecting food production in the tropics and subtropics [1]. It is caused by different species of the genus brucella [2]. The six classical species are *B. abortus* in cattle, *B. melitensis* in goats, *B. suis* in pigs, *B. canis* in dogs, *B. ovis* in sheep, and *B. neotomae* in rat [3–5]. *Brucella abortus*, *B. melitensis*, *B. suis*, and to some extent, *B. canis*, are responsible for the majority of infections in animals and humans [2, 5]. Brucella species are facultative intracellular pathogens that can survive, multiply, and persist within phagocytic cells of the host resulting in lifetime carriage of the organism [6]. Then, ultimately, they become sequestered within monocytes and macrophages of the reticuloendothelial system (RES), such as the lymph nodes, liver, spleen, and bone marrow [7]. Diseased animals shed the pathogen in uterine discharge, vaginal discharge, and milk [8], and these bacteria can spread within the herd through ingestion of contaminated material [9].

Transmission of bovine brucellosis occurs through inhalation, ingestion, and skin abrasions. Cattle become infected after the ingestion of milk from infected cows, food, water, or grazing forage; close contact with infected animals; contact with uterine secretions or aborted fetuses; and through vertical and sexual transmission [10, 11]. Humans are generally infected in one of three ways: eating or drinking something that is contaminated with the bacteria, breathing in the presence of organisms (inhalation), or having the bacteria enters the body through skin abrasions [12–15].

Bovine brucellosis mainly affects sexually mature animals [8, 16, 17], and it is a main cause of reproductive losses, abortion, placentitis, epididymitis, and arthritis in cattle. Adult male cattle may develop orchitis and may result in infertility in both sexes [18–20]. Hygromas, usually involving leg joints, are a common manifestation of bovine brucellosis and may be the only pathognomonic sign of the infection [19]. The clinical manifestations most commonly encountered in humans are relapsing fever, fatigue, malaise, chills, sweats, headaches, myalgia, arthralgia, and weight loss [21–26].

Diagnosis of bovine brucellosis is based upon the isolation of *B. abortus* [16, 17] from abortion material, milk, or necropsy material and serological responses to Brucella antigens [17]. Diagnosis at the herd level as part of eradication schemes has largely relied upon serological tests of biological materials such as milk, serum, vaginal mucus, and semen [27]. Methods of prevention of bovine brucellosis mainly depend on health education to reduce occupational and food-borne risks as well as elimination of the infection among animals through combination of vaccination of all breeding animals to reduce the risks of abortion and raise herd immunity, followed by elimination of infected animals or herds by segregation and slaughter [28, 29].

Although the livestock sector in Ethiopia has a significant contribution to the national economy, productivity (meat and milk) per animal is very low, majorly due to technical constraints and disease like brucellosis [30]. Cross-breeding indigenous cattle with high yielding exotic cattle is the main policy established by the Ethiopian government to bridge the gap between supply and demand for dairy products. Hence, owners of dairy cattle and institutions promoting the dairy industry require current, reliable, and scientific data on such important diseases like brucellosis [31]. Furthermore, brucellosis is a public health problem with adverse health implications both for animals and human being as well as economic implications for individuals and communities even if economic and public health burden of the disease was not investigated in Ethiopia. Management, animal movement, wide ranges of host, herd size, and commingling of different animal species are risk factors for animal brucellosis. The possible risk factors for human brucellosis are feeding behavior, occupational exposure, contact with diseased animals or their products, and discharges [32].

Ethiopia has the second highest burden of zoonotic diseases in Africa [33]. In September 2015, the CDC (Center of Disease Control and prevention) through the GHSA (Global Health Security Agenda) supported the Ethiopian government in prioritizing the zoonotic disease based on severity of disease in humans, proportion of human disease attributed to animal exposure, burden of animal disease, availability of interventions, and existing intersectoral collaboration [34]. Hence, brucellosis was categorized under tier one zoonotic diseases [34, 35]. The disease is known to be an endemic [25] and a growing problem in domestic livestock herds in Ethiopia [19] causing significant loss of productivity through abortion, prolonged calving, kidding, or lambing interval, low herd fertility, and comparatively low milk production in farm animals [36], as well as chronic and febrile illness in humans [37]. An initiative called GHSA addressed the burden of zoonotic diseases like brucellosis and planned to eliminate the disease in five years (between 2017 and 2022) [34]. However, there are no feasible intervention mechanisms currently undergoing in Ethiopia.

Since the first report of livestock brucellosis in Ethiopia by Domenech, [38], the disease has been noted as one of the important livestock diseases in the country [2, 31, 39–42]. Although many reports of seroprevalence of bovine brucellosis are available in Jimma zone, there is no ample information on bovine brucellosis across various livestock production systems (extensive, semi-intensive, and intensive) which gave impetus to the initiation of this study. Hence, currently available information needs to be updated on the status of bovine brucellosis. Therefore, because of these scenarios, this study was conducted with the objectives of studying epidemiological aspects (seroprevalence and associated risk factors) of bovine brucellosis in selected districts of Jimma zone, south western Oromia, Ethiopia.

## 2. Materials and Methods

### 2.1. Description of the Study Areas and Period

**Figure 1:**
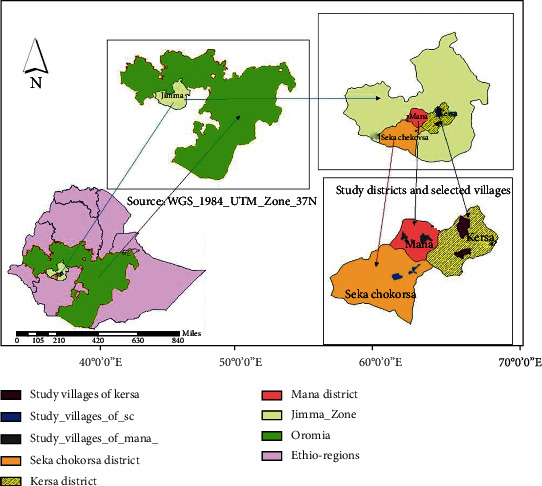
Map of the study areas

The study was conducted at selected districts of the Jimma zone. Jimma zone is geographically located at the South western direction of the country with the distance of 346 km from the capital city, Finfinne (Addis Ababa), having elevation ranging from 880 up to 3360 meters above sea level with 7° 40′-80 2′N latitude and 35° 85′-370 62′ E longitude being categorized as a humid tropical climate with a heavy annual rainfall that ranges from 1200 to 2000 mm that comes from the long and short rainy seasons. The mean annual minimum and a maximum temperature range from 7 to 12°C and from 25 to 30°C [2]. Jimma zone consists of 21 districts and one town administration. Out of them, this study was performed at three districts namely Kersa, Mana, and Seka Chokorsa districts (Figure 1) (which were predetermined by Jimma zone livestock resource development office and Bedelle Regional Veterinary Laboratory Center managements) depending on the monthly report made from respective veterinary clinics. Comparisons of the study districts were described below (Table 1). The study was conducted between the periods of March to August 2021.

### 2.2. Study Design and Sampling Techniques

The study was implemented to assess the prevalence and associated risk factors of bovine brucellosis in the study areas using a cross-sectional study design. Multistage sampling techniques were employed in the present study. A simple random sampling strategy was used for the sampling of the study villages, households (herds), and individual cattle.

### 2.3. Study Populations

The target populations were cattle of different categories of breed, age, and parity kept under intensive, extensive, and semi-intensive management systems at the study districts. As there is no history of vaccination against brucellosis in Ethiopia, all cattle older than six months were included in the study as the risk of the disease is not frequent in cattle of age less than 6 months due to maternal antibodies in the sampling frame. The cattle under study were categorized into two age groups: young (6-24 months) and adult (>24 months) depending on their dentition categorized by Parish and Karisch [43]. All households that allowed blood sample collection from their cattle were used for the analysis of risk factors of bovine brucellosis.

### 2.4. Sample Size Determination

In Jimma zone, there are some previous reports of bovine brucellosis from different districts. From those previous reports, the finding of 6.39% (3.86-8.92 with 95% CI) reported by Tokon et al., [44] from Seka Chokorsa was used for sample size determination due to its recentness and large prevalence as well as inclusion of the district in the present study. Depending on this scenario, 8.92% prevalence was used for sample size calculation according to the sample size calculation recommended by Arya et al. [45], which was the use of previous prevalence result value nearest to 50% to increase the representativeness of the samples and compensation of nonresponsiveness due to withdrawal of response before end of the interview. Therefore, by using the sample size determination formula recommended by Thrusfield [46], the number of samples to be collected was calculated by the following formula:

(1) $$\begin{array}{c} n=\frac{z^{2}*P\exp\left(1-P\exp\right)}{d^{2}}, \end{array}$$

where *n* is the required sample size, *z* is the selected critical value of desired confidence level, *P*exp is the expected prevalence, and *d* is the desired absolute precision. Accordingly, the sample size of the cattle of the study areas to be sampled was calculated by using the expected prevalence of 8.92% at a 95% confidence level and 5% required precision [46].

(2) $$\begin{array}{c} n=\frac{z^{2}*P\exp\;\left(1-\text{Pexp}\right)}{d^{2}}=\frac{\;1.962*0.0892\;\left(1-0.0892\right)}{\left(0.05\right)2}=125\text{ samples for each districts}. \end{array}$$

Accordingly, the calculated sample size from the three study districts was 375; but, to increase the accuracy of the result, the formula recommended by Whitley and Ball [47] which is *N*^″^ = *N*/1 − *q*, where *N*^″^ is the final sample size to be collected, *N* is the first sample size calculated by Thrusfield et al. [46], and *q* is the proportion of attrition in which 11.6% was used. Accordingly, 424 animals were involved in blood sample collection for the study of seroprevalence. But, to maintain representativeness and proportionality of the samples, 140, 114, and 170 blood samples were collected from Kersa, Mana and Seka Chokorsa districts, respectively, depending on their cattle population data shown in Table 1.

For the assessment of risk factors, the sample size was calculated by the formula recommended by Arsham [48]. According to the formula (*N* = 0.25/(SE)2), where *N* is the sample size and SE represents a standard error, the total number of households or livestock owners to be included in the study were 100 by assuming the standard error of 5% at a precision level of 5%, and the confidence interval of 95%. However, to increase the accuracy of the result, the Whitley and Ball [47] formula was used so that a total of 118 livestock owners participated in the questionnaire survey by using a 15.3% for proportion of respondents that were expected to refuse to participate or to drop out before the study ends. But, complete data were collected only from 114 households (41, 28 and 45 from Kersa, Mana, and Seka Chokorsa districts, respectively, based on population data provided in Table 1) because of withdrawal of response before the end of the study. Four respondents were excluded from data analysis due to incomplete information.

## 3. Data Collection

### 3.1. Blood Sample Collection and Testing

For the evaluation of the prevalence of bovine brucellosis in the study areas, 5-7 ml of blood samples were collected aseptically from the jugular vein of individual animals selected for serological examination by using plain vacutainer tubes [18]. The identification number of each of the animals was labelled on corresponding vacutainer tubes. The collected blood samples were kept overnight to allow clotting in slant position at room temperature, and then, the sera were carefully decanted into 1.8 ml labelled cryovials without mixing with the clotted blood [18] at veterinary clinics of respective study districts. The harvested sera were then transported to Bedelle Regional veterinary laboratory center via icebox and stored at -20°C until further processing was held. Blood samples were collected from intensive (46 animals), extensive (363 animals), and semi-intensive (15 animals) management systems depending on the availability of the animals.

Sera samples were tested using a C-ELISA (competitive enzyme-linked immunosorbent assay) (SVANOVIR, Brucella Ab C-ELISA) as indicated by the manufacturer. Initially, the washing solution was reconstituted as directed by the manufacturer. Briefly, PBS-Tween (phosphate-buffered saline) Solution 20x concentrates 1/20 was diluted in distilled water. Then, 500 ml were prepared by adding 25 ml PBS-Tween solution to 475 ml distilled water and mixed thoroughly. Test serums were added per each well of the microtiter plate (wells) in addition to different solutions. The OD (optical densities) was read at 450 nm in a microplate photometer according to the manufacturer's manual. The laboratory test was demonstrated at Bedelle Regional Veterinary Laboratory Center.

### 3.2. Survey for Risk Factor Evaluation

For investigation of determinant factors of bovine brucellosis, general information such as specific location of the animals (districts and villages); breed (local and cross) and age of animals (young, adult); herd size (≤5, 6-10, >10); parity (monoparous and multiparous), reproductive category (bull, heifer, cow); status of pregnancy (pregnant, not pregnant); history of abortion (yes, no); history of retained fetal placenta (yes, no); history of repeat breeding (yes, no); contact with other animal species (yes, no); sources of water for the animals (tap water, underground, surface water or any available water); and feeding style (grazing separately, mixed with other livestock) of the selected animals were documented. For this study, 18 pretested (the questionnaire was tested at 10 arbitrarily selected respondents to check for dialects and confusion or easy understanding of the questionnaire before actual data collection) semistructured questionnaire surveys were answered by all eligible households that their animals were included in the sampling unit for the prevalence study, irrespective of their gender and educational status to investigate risk factors for the occurrence of bovine brucellosis in the study areas. The data were collected by the researcher by face to face interview with the respondents.

### 3.3. Data Analysis

All collected data were entered into Microsoft excel spread sheet version 2010. Then, the data were checked for any kind of errors and correction proceeded if any. Statistical Package for Social Sciences, currently known as Statistical Product for Service Solutions (SPSS) version 20.0 (SPSS Inc., IBM, Chicago, Illinois, USA), was used for statistical analysis of the data. Descriptive statistics like frequency and proportion were employed for the description of the prevalence of the disease and analysis of demographic characteristics of respondents involved in the study. A herd, defined as the total number of cattle belonging to the same household, was considered seropositive if it included at least one seropositive animal. A herd level and individual animal seroprevalence were calculated by dividing the number of positive test results by the total number of herds and animals sampled, respectively.

Univariable analysis using chi-square test was used for the analysis of the association between seropositivity and risk factors associated with the disease. Furthermore, a multivariable logistic regression model was used to analyze risk factors of the disease that was found statistically significant when using univariable analysis and the results were reported by odds ratio using 95% confidence interval to assess the strength of the association. Multivariable logistic regression model selection was based on *p* value (*p* value ≤ 0.25) [46] and backward elimination procedure. The statistical significance level was set at 95% confidence level and 5% level of precision so that *p* value ≤ 0.05 was considered significant. The model validity and predictive ability were assessed using the Hosmer-Lemeshow test.

## 4. Results

### 4.1. Seroprevalence

The overall seroprevalence of bovine brucellosis in the present study areas was found to be 3.3% and 12.3% at the animal and herd level, respectively, from which the highest prevalence was detected in Kersa district with a proportion of 6.4% at animal level and 22% at herd level (Table 2). The sociodemographic profile of the respondents indicated that 94 (82.5%) were males and 20 (17.5%) were females from which 68 (59.6%) were found between the age of 41 and 60 years while 45 (39.5%) attended basic education (Table 3).

### 4.2. Risk Factor Evaluation

Analysis of some host risk factors and seroprevalence of bovine brucellosis showed that all of the animals tested positive were adult as well as local breeds whereas 11 (3.6%) were female from which 9 (4.9%) and 2 (3.6%) cows were multiparous and monoparous, respectively. On the other hand, body condition, status of pregnancy, history of abortion, and history of repeat breeding were found statistically significant by multivariable logistic regression model (*p* value < 0.05) (Table 4). Analysis of management risk factors and seroprevalence indicated that all seropositive animals were managed under an extensive management system, had frequent contact with other herds or flocks, and had no separate parturition pen for pregnant animals in which 6 (13.6%) and 8 (16.7%) seropositive animals were from small and medium herd sizes. The feeding styles of 12 (22.2%) of seropositive animals were mixed with other herds and found statistically significant by multivariable logistic regression analysis (*p* value = 0.007) (Table 5).

## 5. Discussion

This study is relatively different from other research conducted at different parts of Jimma zone in that it included all management systems (extensive, semi-intensive, and intensive management systems) where several researches conducted in this study area were done exclusively by considering specific management systems. The overall seroprevalence of bovine brucellosis in the current study was 3.3% (95% CI: 1.82-5.48) at the individual animal level (Table 2). In line with this result, the previous reports of 3.19% by Berhe et al. [49] in Tigray region, 3.1% by Ibrahim et al. [50] in Jimma zone, 3.5% by Megersa et al. [51] in Southern and Eastern Ethiopia, 3.2% by Asmare et al. [52] in central and southern Ethiopia, 1.97% by Degefu et al. [53] in east Wollega zone, 2.6% by Asmare et al. [2] on exotic and cross bred cattle in dairy and breeding farms, 2.4% by Asgedom et al. [54] from Alage district, 3.23% by Geresu et al. [31] in Asella and Bishoftu towns, 2.6% by Tsegaye et al. [55] in Arsi Zone, 3.75% by Waktole et al. [56] in selected dairy farms of Bishoftu town, 3.65% by Bulcha et al. [42] in and around Adama Town, and 3.0% of pooled seroprevalence by Dejene et al. [57] in Ethiopia had nearly similar animal level seroprevalence. Likewise, the reports from other African countries have shown nearly similar results. For instance, 3.3% by Nakoune et al. [58] in Central African Republic and 3.4% by Ndukum et al. [59] from cattle selected in different areas in Cameron.

In comparison with this finding, the relatively lower seroprevalence of 1.7%, 0.2%, and 1.04% were reported by Tschopp et al. [60] in Arsi zone, Bashitu et al. [19] in Debrebirhan and Ambo Towns, and Tadesse et al. [61] in Becho District, South West Shewa, respectively. However, higher seroprevalence reports were made by Megersa et al. [62] (10.6%) in Borena zone and Negash and Dubie [63] (5.7%) in Afar region. Similarly, relatively higher results of seroprevalence were reported in other African countries; Matope et al. [64] with 5.6% in Zimbabwe, Mensah et al. [65] with 21.9% in Ghana, and Mai et al. [66] with 24.0% in Nigeria. The variation in prevalence might be due to differences in the study population, study protocol, agroecology, and differences in diagnostic tests applied among different researches [62, 67].

In the present finding, the district-related seroprevalence showed that the highest positive reactors were recorded in Kersa district with a proportion of 6.4% (95% CI: 2.90-11.85) followed by 1.76% (95% CI: 0.37-5.07) in Seka Chokorsa and 1.75% (95% CI: 0.21-6.20) in Mana districts (Table 2). These results vary from the previous reports of the three districts. For instance, Tolosa [68] found no seropositive reactors in all the three districts. However, Tokon et al. [44] reported 6.39% in Seka Chokorsa district. The variation across the different research in the districts may be due to variation in the age and sex, physiological status of animals involved in the study, and breakdown of hygienic practices in and around the study districts. Although the finding of Dirar et al. [7] reported no seroprevalence of bovine brucellosis in Jimma town, the report of 0.2% and 1.16% by Tolosa [68] in Jimma town and Dedo district and 6.39% and 5% by Tokon et al. [44] in Seka Chokorsa and Shebe Sombo districts indicated the circulation of the bacteria in the areas, so that high probability of transmission and spread into adjacent districts like Kersa, Mana and Seka Chokorsa.

On the other hand, the current finding also showed that the herd level seroprevalence was 12.3% (95% CI: 6.88-19.75) (Table 2) which is slightly concordant with the report of 11.2% by Dinka and Chala [69] in pastoral and agropastoral areas of East Showa Zone, 13.6% by Jergefa et al. [70] in central Oromia, and 11.6% by Robi and Gelalcha [71] in breeding female cattle under the traditional production system of Jimma zone, but found lower than the finding of 26.1% by Megersa et al. [51] in Southern and Eastern Ethiopia, 25.8% by Abera et al. [32] in Hawassa Town, and the reports of other African countries such as Uganda (55.5%) by Faye et al. [72] and Zambia (61%) by Muma et al. [73], but higher than that of 2.96% by Tolosa [68] in Jimma zone and 4.9% by Agga et al. [74] in western Ethiopia. Such contrasting findings may be related to the overall individual animal level prevalence status, the size of studied herds, and the difference in management systems and herd sizes among animals involved in the studies [2].

Concerning breed susceptibility to brucellosis, the present study revealed that all the seropositive cattle were local breeds and none of the cross-breed cattle found seropositive (Table 4). But this does not mean that the disease is insignificant in cross-breed as it is a very serious disease responsible for reproduction failure and economic loss in the dairy industry [19]. Rather, seronegativity in cross-breed in this study might be due to the origin of the animal from the previously uninfected or unexposed herds [75]. Similar to this result, Bulcha et al. [42] reported that all the seropositive animals were local breeds. In the same manner, Terefe et al. [11], Abera et al. [32], and Robi and Gelalcha [71] reported higher seroprevalence of bovine brucellosis in local breeds of cattle. However, contrary to the current study, Eticha et al. [75], Abera et al. [32], and Teka et al. [76] reported higher seroprevalence of brucellosis in cross-breed than in local breeds. This variation may be due to variation in the breeds of animals sampled, management practice and herd size, better management in the cross herds, and separate feeding that minimize contacts between animals.

In the present finding, the body condition had shown significant association with the seroprevalence of bovine brucellosis. Hence, out of the total of seropositive cattle, 10 (7.3%) were in poor body condition, whereas 3 (1.6%) were in medium and the rest 1 (0.9%)) were in good body condition. Multivariable logistic regression analysis result indicated that seropositivity is 4.8 (AOR = 4.8 with 95% CI: 2.00-22.74) and 2.7 (AOR = 2.7 with 95% CI: 1.10-5.26) times more likely common in poor and medium body condition of cattle when compared with good body condition (Table 4). In accordance with this finding, Ejeta et al. [40] and Abera et al. [32] reported high positive reactors in poor body condition cattle than in medium and good body condition, but Ndukum et al. [59] and Robi and Gelalcha [71] reported higher seroprevalence in good body condition than poor body condition. High seroprevalence in poor body condition animals might be due to, most probably; poor body condition animals are allowed free grazing comingling with other animals that increase the risk of exposure to bovine brucellosis. On top of this, because of scarce resources, animals that are not well fed or malnourished may be stressed and immunosuppressed predisposing them to the disease [77].

On the other hand, 7 (8.2%) seropositive animals were pregnant whereas the remaining 4 (2.6%) were nonpregnant whereby statistically significant association has been observed (*p* value = 0.009) (Table 4) in which pregnant cows were 4.3 (AOR = 4.3 with 95% CI: 1.43-13.04) times more likely to be seropositive than nonpregnant cows. This finding is in agreement with the report of Haileselassie et al. [78] and Teka et al. [76] who reported high brucella-positive reactors in pregnant cows. Likewise, Tulu et al. [79] reported that seropositivity was 3 times more likely common in pregnant cows than nonpregnant and the association was found statistically significant. However, Tsegaye et al. [55] and Robi and Gelalcha [71] reported high seroprevalence of brucellosis in nonpregnant cows than in pregnant cows. Bovine brucellosis is essentially a disease of sexually mature animals and susceptibility increases with sexual maturity and pregnancy [2, 52] due to the influence of sex hormones and placental erythritol sugar that facilitate the pathogenesis of brucellosis [80].

In this study, analysis of the risk factors associated with the previous history of cows indicated that 7.8% have encountered abortion at least once in their lifetimes and the odds of bovine brucellosis were 3.3 (AOR = 3.3 with 95% CI: 1.07-10.21) times more likely common in cows with a history of abortion showing statistically significant association (*p* value = 0.038). Likewise, 6.6% were seropositive among the cows with history of repeat breeding. According to this result, seropositivity is 2.7 (AOR = 2.7 with 95% CI: 1.86-8.15) times more likely common in animals with repeat breeding having a statistically significant association (*p* value < 0.001) (Table 4). In agreement with this finding, Agga et al. [74], Geresu et al. [31], Tsegaye et al. [55], and Jatana [81] reported the association between brucellosis seroprevalence and occurrence of abortion. In the same manner, Bashitu et al. [19] reported a statistical association of history of abortion and the presence of infection in animals. However, according to the report of Segwagwe et al. [82], seropositivity was highly associated with nonaborted cows than aborted cows. This variation may be resulted from discrepancies in number of animals involved, the sources from which the cows were bought and management practices.

In the present study, all seropositive animals were managed under extensive management system (Table 5). In line with this result, Alem and Solomon [83], Belihu [84], Segwagwe et al. [82], and Teka et al. [76] were unable to find positive reactor in intensive dairy farms in Fafan Zone of Ethiopian Somali and central Ethiopia, in intensive dairy farms in Addis Ababa area, Nyagatare District of Rwanda, and Becho district, south west Shewa, respectively. In contrary to this report, Geresu et al. [31] reported higher brucella seropositive reactors in intensive production systems than extensive and semi-intensive production. The main reason for higher seroprevalence in the present study might be due to free movement of animals, purchase of infected cattle from unknown source, wildlife interaction, use of common pastures and water sources, mixing with other livestock, and variation of the number of animals included [2, 66].

In this study, the frequent contact with other livestock analysis indicated that all of the brucella-positive reactors had frequent contact with other herds or flocks (Table 5). In the same manner, Robi and Gelalcha [71] and Tulu et al. [79] reported higher seroprevalence in mixed herds with other livestock than separate cattle herd. Moreover, Al-Majali et al. [85] in Jordan, Megersa et al. [62] in Borena, and Anka et al. [86] in Malaysia reported mixing of sheep and/goats with cattle increased risk of brucella seropositivity in bovine. Given that contacts between cattle, sheep, and goats are the most important risk factor, the control of movements of infected sheep and goats as well as control of brucellosis in the later species may reduce seroprevalence and spread of *B. melitensis* in cattle in mixed herds [2, 4]. Such variation across different reports could be due to differences in environmental factors, animal breed, and management practices [71].

Depending on multivariable logistic regression analysis of the feeding styles, 2 (3.3%) and 12 (22.2%) of seropositive animals were fed by being separated and mixed with other livestock species, respectively. Out of management risk factors considered in this study, feeding style was found statistically significant by multivariable regression analysis (*p* value = 0.007). According to this result, mixed feeding style was 8.3 (AOR = 8.3 with 95% CI: 1.76-38.99) times more likely risky than separate grazing (Table 5). This may be due to the fact that, through mixed grazing, brucella species can be transmitted from livestock species to the other and even within the same species during feeding. Therefore, separate grazing is highly recommended to fight against bovine brucellosis.

Regarding the origin history of animals involved in this study, more than half (8 (20.5%)) of the sources of replacement stock of seropositive animals were from market whereas the rest 6 (0.1%) of them were from mixed sources (Table 5). In line with this result, Asmare et al. [2], Teka et al. [76], and Gugsa et al. [87] reported high positive reactors in purchased animals, but the report of Tesfaye et al. [88] showed high brucella seroprevalence in both purchased and home-bred animals. These animals might be purchased from herds infected with bovine brucellosis. This indicates outside sources for stock replacement could be one possible way of introducing the disease into unaffected farms because of loose biosecurity [87]. Herds receiving purchased cattle from other farms have high odds of brucella infection through the introduction of infected cattle [73]. This could be the result of a lack of awareness by the livestock owners buying the defective cow and the absence of regulatory imposition in the system [2].

On the other hand, the sources of water for the seropositive herds in this study were underground water 2 (15.4%), surface water 3 (23.1%), both underground and surface water 5 (12.5%), and available water 4 (18.2%) whereas no brucella-positive reactor was found in the herds provided with tap water (Table 5). The result obtained from this study indicated that water could be predisposing factor to bovine brucellosis because of contamination of water sources by brucella-infected materials such as aborted fetuses and retained fetal placenta that are dumped into the environment so that draining the materials in to water sources through flooding. Likewise, lack of clean drinking water for animals is positively associated with seropositivity [89]. Moreover, contact of different animals sharing the same water sources might be the major mechanism in which brucella is transmitted and spread across different animals.

Bovine brucellosis is a zoonotic disease of humans and animals covering wide geographic areas of the world particularly developing countries [4, 90]. In Ethiopia, several seroprevalence of the disease have been investigated including the current study. Effective control strategies of bovine brucellosis consist of surveillance, prevention of transmission, and controlling the reservoir of infection by different methods including culling [91, 92]. Investigation of seroprevalence of a disease gives a foundation for the establishment of control and prevention strategy in a given country to minimize economic and public health burdens of the disease so as to increase livestock production and productivity as well as protection of human health and welfare.

## 6. Conclusion

From this study, it can be concluded that bovine brucellosis was found prevalent in the current study areas with highest seroprevalence in Kersa district. Risk factors such as body condition, status of pregnancy, history of abortion, and repeat breeding, as well as feeding style, had been found significantly associated with the occurrence of the disease. Therefore, much attention should be given to these potential risk factors in order to establish and implement proper prevention and control strategies of bovine brucellosis so as to prevent possible human health hazards and economic deterioration due to the disease.

### 6.1. Limitation of the Study

Blood samples examined in this study did not utilize screening test like Rose Bengal Plate test due to inaccessibility of the kit to the required amount so that C-ELISA was used for all of the samples.

## Figures and Tables

**Table 1:** Description of the study districts.

Geographical characteristics	Study districts		
	Kersa	Mana	Seka Chokorsa
Latitude	7° 58′-80 02′	7° 66′-7° 91′	7° 30′-7° 76′
Longitude	36° 73′-37° 24′	36° 60′-36° 88′	36° 27′-36° 84′
Total cattle population	198,084	151,289	217,689
Total number of households	27,927	20,875	32,006
Number of villages	34	26	36

Sources: Livestock Resource Development offices of respective districts, 2020 (unpublished data).

**Table 2:** Results of C-ELISA across study districts and villages.

Study districts	Towns and villages	Animal level seroprevalence		Herd level seroprevalence	
		**N** (+ve)	Prevalence (95% CI)	**N** (+ve)	Prevalence (95% CI)
Kersa	Serbo	46 (3)	6.5 (1.37-17.90)	14 (3)	21.4 (4.66-50.80)
	Tikur Balto	43 (3)	7 (1.46-19.06)	13 (3)	23.1 (5.04-53.81)
	Wayu	51 (3)	5.9 (1.23-16.24)	14 (3)	21.4 (4.66-50.80)

Over all result		140 (9)	6.4 (2.90-11.85)	41 (9)	22 (10.56-37.61)

Mana	Bilida	41 (1)	2.4 (0.06-12.86)	10 (1)	10.0 (0.25-44.50)
	Haro	27 (1)	3.7 (0.09-18.97)	8 (1)	12.5 (0.32-52.65)
	Yebu	46 (0)	0	10 (0)	0

Over all result		114 (2)	1.75 (0.21-6.20)	28 (2)	7.1 (0.88-23.50)

Seka Chokorsa	Buyo Kachema	35 (1)	2.86 (0.07-14.92)	12 (1)	8.3 (0.21-38.48)
	Seka	71 (2)	2.82 (0.34-9.80)	19 (2)	10.5 (1.30-33.14)
	Shashemenne	64 (0)	0	14 (0)	0

Over all result		170 (3)	1.76 (0.37-5.07)	45 (3)	6. 7 (1.40-18.27)
Over all total		424 (14)	3.3 (1.82-5.48)	114 (14)	12.3 (6.88-19.75)

CI: confidence interval; *N*: frequency.

**Table 3:** Sociodemographic characteristics of households involved in the study.

Variables	Category	Kersa (%)	Mana (%)	Seka Chokorsa (%)	Total (%)
Gender	Male	34	22	38	94 (82.5)
	Female	7	6	7	20 (17.5)

Age category	18-25	5	3	6	14 (12.3)
	26-40	5	4	6	15 (13.2)
	41-60	24	18	26	68 (59.6)
	>60	7	3	7	17 (14.9)

Educational status	Illiterate	11	8	13	32 (28.1)
	Basic education	17	8	20	45 (39.5)
	Primary	10	10	8	28 (24.6)
	High school	3	2	4	9 (7.9)

Marital status	Single	5	3	6	14 (12.3)
	Married	32	24	35	91 (79.8)
	Divorced	1	0	3	4 (3.5)
	Widowed	3	1	1	5 (4.4)

**Table 4:** Association of the seroprevalence of bovine brucellosis across host-related risk factors.

Risk factors	Category	**N** (+ve)	Prevalence (%)	Univariable analysis		Multivariable analysis	
				*χ* ^2^	**p** value	AOR (95% CI)	**p** value
Age	Young	106 (0)	0	4.826	0.028		
	Adult	318 (14)	4.4				
Sex	Male	121(3)	2.5	0.359	0.549		
	Female	303 (11)	3.6				
Breed	Local	349 (14)	4.0	3.111	0.078		
	Cross	75 (0)	0				
Body condition	Poor	137 (10)	7.3	10.272	0.006	4.8 (2.00-22.74)	0.005
	Medium	186 (3)	1.6			2.7 (1.10-5.26)	0.016
	Good (ref)	106 (1)	0.9				
Parity	Monoparous	55 (2)	3.6	6.198	0.05		
	Multiparous	185 (9)	4.9				
Status of pregnancy	Yes	85 (7)	8.2	8.104	0.004	4.3 (1.43-13.04)	0.009
	Not (ref)	155 (4)	2.6				
Reproductive category	Bull	121 (3)	2.5	3.642	0.162		
	Heifers	63 (0)	0				
	Cows	240 (11)	4.6				
History of abortion	Yes	64 (5)	7.8	4.803	0.028	3.3 (1.07-10.21	0.038
	No (ref)	176 (6)	3.4				
History of RFP	Yes	84 (6)	7.1	0.024	0.877		
	No (ref)	156 (5)	3.2				
History of repeat breeding	Yes	76 (5)	6.6	3.115	0.078	2.7 (1.86-8.15)	<0.001
	No (ref)	164 (6)	3.7				

AOR: adjusted odds ratio; *χ*^2^: chi square; CI: confidence interval; *N*: number of observation; RFP: retained fetal placenta.

**Table 5:** Influence of management risk factors on seroprevalence of bovine brucellosis.

Risk factor	Category	**N** (+ve)	Prevalence (%)	Univariable analysis		Multivariable analysis	
				*χ* ^2^	**p** value	AOR (95% CI)	**p** value
Management systems	Intensive	14 (0)	0	4.256	0.119		
	Extensive	90 (14)	15.6				
	Semi-intensive	10 (0)	0				

Herd size	Small	44 (6)	13.6	4.012	0.135		
	Medium	48 (8)	16.7				
	Large	22 (0)	0				

Frequent contact with other herds	Yes	98 (14)	14.3	2.606	0.016		
	No	16 (0)	0				

Feeding style	Separate (ref)	60 (2)	3.3	9.413	0.002		
	Mixed	54 (12)	22.2			8.3(1.76-38.99)	0.007

Source of replacement stock	Market	39 (8)	20.5	4.843	0.089		
	Own	15 (0)	0				
	Both	60 (6)	0.1				

Type of service	AI	42 (3)	7.1	0.306	0.858		
	Bull	6 (0)	0				
	Both	66 (8)	12.1				

Types of the housing system	Loose	15 (1)	6.7	0.505	0.477		
	Tying	99 (13)	13.1				

Sources of water	Underground	13 (2)	15.4	5.876	0.209		
	Surface	13 (3)	23.1				
	Both	40 (5)	12.5				
	Tap water	26 (0)	0				
	Any available	22 (4)	18.2				

Separate parturition pen	Yes	7 (0)	0	0.732	0.392		
	No	105 (14)	13.3				

AI: artificial insemination; AOR: adjusted odds ratio; *χ*^2^: chi square; CI: confidence interval; *N*: number of observation.

## Data Availability

All data supporting these research findings are included within the manuscript. The databases (without personally identifiable information) are available from the corresponding author upon request.

## Abbreviations

AOR: Adjusted odds ratio
CI: Confidence interval
C-ELISA: Competitive enzyme-linked immunosorbent assay
FAO: Food and agricultural organization
OD: Optical densities
OIE: World Organization for Animal Health
PBS: Phosphate-buffered saline.
